# Osteochondral tissue repair in osteoarthritic joints: clinical challenges and opportunities in tissue engineering

**DOI:** 10.1007/s42242-018-0015-0

**Published:** 2018-05-28

**Authors:** Maryam Tamaddon, Ling Wang, Ziyu Liu, Chaozong Liu

**Affiliations:** 1 Institute of Orthopaedic and Musculoskeletal Science, Division of Surgery and Interventional Science, University College London, Royal National Orthopaedic Hospital, Stanmore, HA7 4LP UK; 2 School of Mechanical Engineering, Xi’an Jiaotong University, Xi’an, 710049 People’s Republic of China

**Keywords:** Osteochondral tissue engineering, Osteoarthritis, Osteochondral scaffold, Cartilage and subchondral bone, Clinical scaffolds

## Abstract

Osteoarthritis (OA), identified as one of the priorities for the Bone and Joint Decade, is one of the most prevalent joint diseases, which causes pain and disability of joints in the adult population. Secondary OA usually stems from repetitive overloading to the osteochondral (OC) unit, which could result in cartilage damage and changes in the subchondral bone, leading to mechanical instability of the joint and loss of joint function. Tissue engineering approaches have emerged for the repair of cartilage defects and damages to the subchondral bone in the early stages of OA and have shown potential in restoring the joint’s function. In this approach, the use of three-dimensional scaffolds (with or without cells) provides support for tissue growth. Commercially available OC scaffolds have been studied in OA patients for repair and regeneration of OC defects. However, none of these scaffolds has shown satisfactory clinical results. This article reviews the OC tissue structure and the design, manufacturing and performance of current OC scaffolds in treatment of OA. The findings demonstrate the importance of biological and biomechanical fixations of OC scaffolds to the host tissue in achieving an improved cartilage fill and a hyaline-like tissue formation. Achieving a strong and stable subchondral bone support that helps the regeneration of overlying cartilage seems to be still a grand challenge for the early treatment of OA.

## Osteoarthritis and advancement in its treatment

In articulating joints, the articular cartilage, calcified cartilage and subchondral bone form a composite system, referred to as the OC unit, which has the unique capability of transferring loads during joint motion [1]. Repetitive overloading to the joint could result in cartilage damage and changes in the subchondral bone, leading to mechanical instability of the joints and loss of joint function [2, 3]. If left untreated, the OC defects will lead to the development of OA [2], where the composition and structure of this unit undergo significant alterations [1]. During the progression of OA, thinning and degradation of articular cartilage, joint-space narrowing, osteophytes formation and subchondral bone remodeling [4–6] take place.

**Fig. 1:**
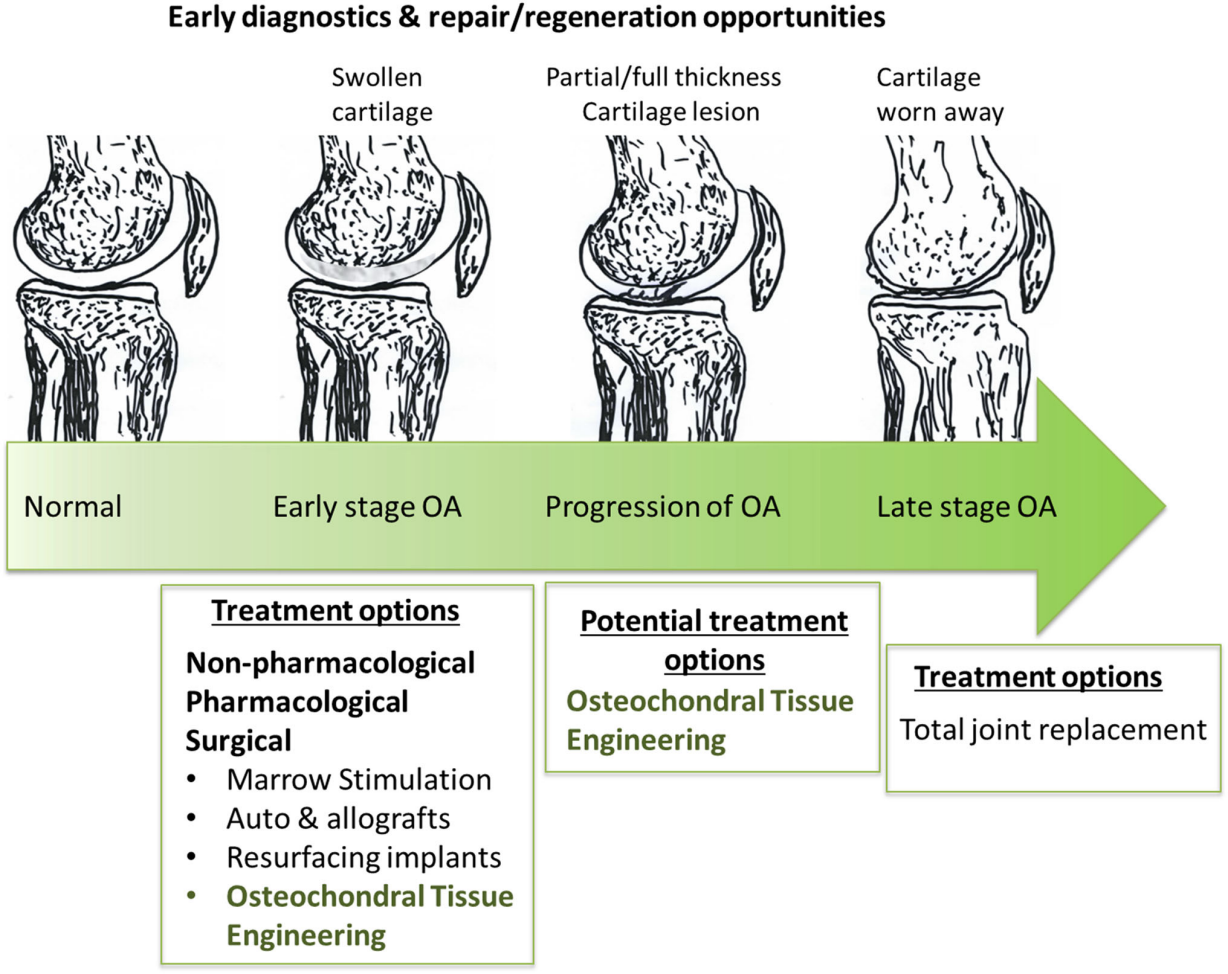
Progression of OA: conditions and treatments in each stage

Other pathological processes including microfractures, microedema or microbleeding within the subchondral bone could lead to subchondral bone defects such as subchondral cyst formation [4]. If the OC defect has progressed to the stage where the patient’s quality of life has significantly reduced and non-surgical treatments are no longer effective, then a joint replacement has to be performed. This major surgical procedure often does not restore the full function of joints and has high long-term complication rates. The process of OA and available treatment options are shown in Fig. 1.

In early stages of OA, pain and stiffness dominate the other symptoms, and the goal of the treatments is therefore to reduce pain and physical disability and some attempt to control structural deterioration in the affected joints [6, 7], using physical therapy [8], analgesics and NSAIDs [9]. Intra-articular injection of long-acting glucocorticoids is an effective treatment of inflammatory flares of OA. Hyaluronic acid has varying effectiveness when used for intra-articular injections for the treatment of OA of the knee [7].

With the progression of OA, where the cartilage defect is still small (area $<2-3\hbox {cm}^{2})$, microfracture (MF) marrow stimulation is considered a medically necessary treatment. MF is a minimally invasive procedure which involves removing the damaged cartilage and then drilling into the surface of the underlying bone in order to allow blood and bone marrow to come through to the bone/cartilage interface, where the mesenchymal stem cells contribute to the formation and repair of the cartilage and bone. However, the regenerated cartilage is mainly fibrocartilage and is not expected to have the same durability as the articular hyaline cartilage. This type of cartilage is mostly type I collagen, fibrocytes and a disorganized matrix that lacks the biomechanical and viscoelastic characteristics of normal hyaline cartilage [10] and can fail with high shear forces in the joint, leading to an ongoing articular surface irregularity and subsequent secondary arthritic changes [11]. This is demonstrated by the high 5-year post-MF re-operation rates, which is between 30 and 50 % [12].

OC autografts or allografts [12], scaffolds and focal knee resurfacing implants are among the approaches that have been explored for treatment of small- to mid-sized lesions [13]. OC autografts have been proposed to provide an immediate reliable tissue transfer of a viable OC unit in a single-stage procedure. This procedure exploits the regenerative potential of bone and bone-to-bone healing, since the cartilage has a limited healing capacity [12].

Fresh OC allografts provide the surgeon with more freedom regarding the size of the defect that can be treated. Common indications for OC allograft include large, focal chondral defect, osteochondritis dissecans and unicompartmental arthritis [14]. However, apart from general complications of open joint surgery, OC allograft transplantation is also associated with a risk of disease transmission from the allograft and subchondral collapse due to inadequate integration. The latter is responsible for a majority of graft-related failures [12].

Tissue engineering (TE) approaches have been developed as potential solutions for repair and regeneration of OC defects as illustrated in Fig. 1. In this approach, scaffolds are designed and fabricated to provide a physical environment to support cellular activities and prompt tissue regeneration. OC scaffolds can be implanted by arthroscopy or mini-arthrotomy and fixed by press fit. Some cases may require additional fixation through sutures, pins or fibrin glue. Currently, lesion size range from 2 to $8~\hbox {cm}^{2}$ can be treated using OC scaffolds which are available in predetermined sizes or patches that can be shaped and sized at the time of implantation [12]. Commercially available scaffolds such as Chondromimetic (Tigenix NV), MaioRegen (FInceramica) and TruFit$\circledR$ BGS Plugs (Smith & Nephew) have been used, with or without cells, in clinical trials for treatment of small cartilage and osteochondral defects (OCDs)($<1.5\, \hbox {cm}^{2})$. However, limited success was reported, and none of these scaffolds have achieved satisfactory durable clinical results.

To date, OC TE approaches have mainly focused on regeneration of small OC defects mostly in early stages of OA. However, with the right scaffold, treatment of large, late-stage OC defects could become possible. The idea of a “smart” scaffold which provides an appropriate biomechanical environment to support healthy cell growth and promote OC regeneration has been reported as the Holy Grail in the last decades in the treatment of both early and late stages of OA. However, this has been achieved only in early stages of OA, and with limited success. In this paper, we discuss the requirements of an OC scaffold, design and fabrication techniques and insights from the studies of OC scaffolds performance in clinical settings, in light of similar events observed during the development of OA. The effect of biomechanical and biological fixations of the scaffold on the healthy regeneration of OC tissue has become increasingly apparent. The results discussed in this study would provide us with the essential knowledge for the successful development of future clinical OC scaffolds.

## Osteochondral tissue engineering

TE is a discipline that applies the knowledge of materials science, cell biology and bioengineering to construct tissue templates and restore the function of an injured tissue. It may involve a cell-free approach by using a scaffold only, or it may involve taking the cells from the patient, seeding the cells onto a scaffold and culture this whole in a bioreactor system, then transplanting it back into the patient once the tissue has matured. In either processes, the three-dimensional porous scaffold plays an important role in supporting the cells growth and guiding new tissue formation [15]. Following the natural structure/composition of the extracellular matrix (ECM), a scaffold intends to regenerate forms the basis of biomimetic scaffolds, and as such, biomimetic OC scaffolds are usually designed in bi- or multilayered structure to mirror the natural tissue structure. Recently, bioporinting techniques have enabled the fabrication of zonal architecture that more closely matches this natural tissue.

### Osteochondral unit

**Fig. 2:**
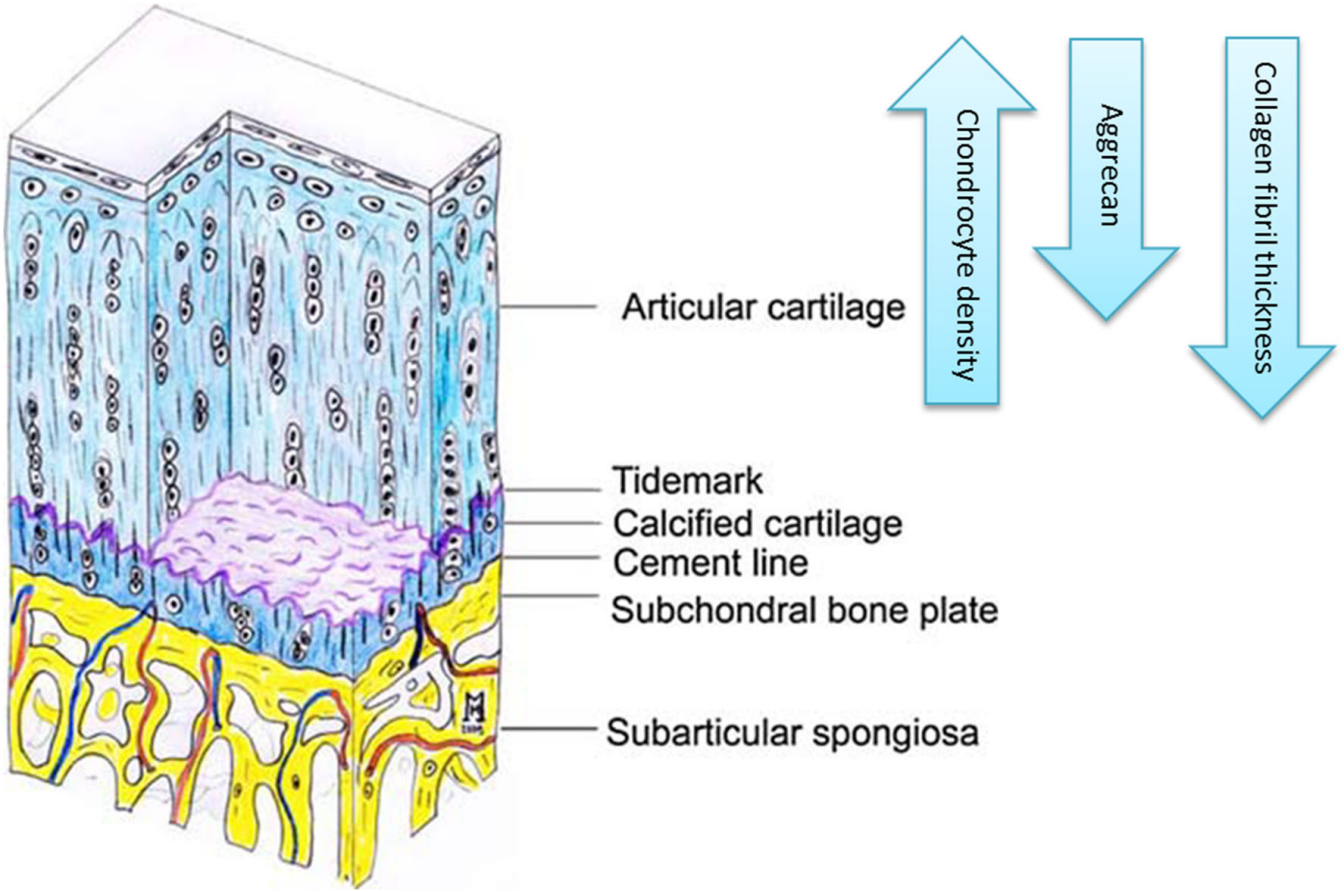
Osteochondral unit: cartilage and subchondral bone. Tidemark denotes a discrete band between mineralized and non-mineralized cartilage [4]. Reproduced with permission from Springer Nature

The OC tissue is composed of cartilage and subchondral bone as shown in Fig. 2, each with its own specific hierarchical structure and biological property [16]. Therefore, to design a biomimetic scaffold an understanding of the OC unit, including its composition, structure and function is essential.

#### Cartilage–bone junction

Articular cartilage, the top layer of an OC unit, is vital for facilitating a smooth motion within joints and absorbing impact. It consists of chondrocytes embedded in an ECM mainly comprising collagen (60% dry weight [17], 90–95% type II [18]), proteoglycans and non-collagenous proteins. Typically, articular cartilage is divided into four zones based on the distance from the surface: superficial, middle, deep and calcified zones [18]. The latter is directly below the deep zone containing hypertrophic chondrocytes embedded in a densely mineralized matrix which constitutes the OC interface [19]. Calcified cartilage is separated from the deep zone by a discrete band of mineralized cartilage called “tidemarks” (see Fig. 2). This line represents the mineralization front of the calcified cartilage and provides a gradual transition between the two dissimilar regions of cartilage (non-calcified and calcified). Immediately below the calcified zone lies subchondral bone plate—a bony lamella (cortical endplate, 1–3 mm thick [20]), which is separated by a “cement line” from the calcified cartilage. Together with the supporting trabeculae and subarticular spongiosa, they form the subchondral bone unit [4], as illustrated in Fig. 2.

#### Role of subchondral bone in maintenance of cartilage

Subchondral bone is essential in function and maintenance of articular cartilage. From biomechanical point of view, subchondral bone enhances the load-bearing capacity by attenuating the majority of load on the joints [4]. Normal subchondral bone attenuates about 30% of the loads through joints; only 1–3% is attenuated through cartilage.

From nutritive point of view, the microvessels extended from subchondral bone to cartilage provide it with essential nutrients [4]. Whereas the superficial zone of cartilage is mainly dependent on diffusion via synovial fluid as its nutritive source, the subchondral circulation may make a significant contribution to the nutrition in deep and calcified cartilage [21, 22]. The abrogation of contact between the subchondral bone and cartilage leads to degeneration of cartilage in the long run [23]. This emphasizes the importance of subchondral bone regeneration and vascularization in OC TE.

Any damage to one of these subunits alters the fine mechanical and biochemical balance that exists within the OC tissue, and if the damage exceeds a critical size usually surgical interventions are required.

### Osteochondral Scaffold design and fabrication techniques

An ideal OC scaffold should provide an appropriate microenvironment for native cells to grow and promote tissue regeneration. In fact, scaffold surface and material characteristics affects cell attachment and differentiation, while scaffold microstructure affects cell adhesion, migration and differentiation.

Biomaterials used in tissue engineering of OCDs are usually categorized into four major groups: natural polymers, synthetic polymers, metallic materials and inorganic materials such as ceramics and bioactive glasses. Multicomponent systems can be designed to generate composites of enhanced performance [2]. Naturally derived polymers are obtained primarily from plants, animals and microbial sources which are again classified based on their chemistry into polysaccharide, protein, polyester, polyamide-based polymers [24]. Natural polymers such as collagen, alginate, gelatin and chitosan have the advantage of native biological function, enhancing cellular attachment, proliferation and function [25, 26]. Cells primarily interact with scaffolds via ligands on the material surface. Scaffolds synthesized from natural extracellular materials naturally possess these ligands in the form of RGD binding sequences, whereas scaffolds made from synthetic materials may require deliberate incorporation of these ligands through, for example, protein adsorption [27]. The main disadvantages of these naturally derived biomaterials are batch-to-batch variability and low mechanical strength. With synthetic polymers (e.g., PCL, PLA, PLGA) on the other hand, it is possible to precisely control the mechanical properties and tailor the structure and apply surface modifications. However, they exhibit poor cell adhesion due to their intrinsic hydrophobicity and lack of natural ligand binding sites [26] .

An important consideration in designing an OC scaffold is the biodegradation rate that should match the rate of new tissue formation [28]. Biodegradation of polymeric biomaterials involves firstly degradation, which is the cleavage of hydrolytically or enzymatically sensitive bonds in the polymer [29, 30] into low molecular weight fractions, and secondly erosion, which is dissolution and diffusion of these low molecular weight fractions [31]. The biodegradation rate of a polymer depends mainly on the intrinsic properties of the polymer [32].

Natural polymers are said to be the first biodegradable biomaterials used clinically. The rate of *in vivo* degradation of enzymatically degradable polymers such as collagen, however, varies significantly with the site of implantation depending on the availability and concentration of the enzymes [30]. Hydrolytically degradable synthetic polymers on the other hand have minimal site-to-site and patient-to-patient variations compared to enzymatically degradable polymers [30, 31]

Bioceramics, such as calcium phosphates, are known for their excellent osteoconductity [2, 33]. The most common types of calcium phosphates for bone TE scaffolds are hydroxyapatite ($$\hbox {Ca}_{10}$$($$\hbox {PO}_{4})_{6}$$(OH)$$_{2})$$, tricalcium phosphate, biphasic calcium phosphates and multiphasic bio-glasses [34]. The physical properties of the calcium phosphate ceramics, such as degradation rate, modulus and processability, can be controlled by altering their composition [35].

In order to provide a suitable microenvironment for the cells, scaffolds need to supply them with a three-dimensional space, proliferate and differentiate into the appropriate tissue type there. Cell migration requires scaffold to be porous [36, 37] and to have an interconnected pore structure to allow for healthy cellular invasion and growth, nutrition delivery [38] to the cells inside the scaffold, as well as removal of metabolic waste from the cells. Vascularization is not therefore possible without porosity to allow oxygen and nutrition diffusion and vasculature formation [39].

Highlighting the significance of vasculature in bone formation is the fact that the metabolically active cells are no more than 100 $\mu$m away from a capillary for supply of oxygen and nutrients [40, 41]. This needs to be taken into account when designing a scaffold for OC TE, for example, by devising internal channels [42] in the OC scaffold.

There are a number of techniques available to produce porous scaffolds, depending on the scaffold material. Pore-inducing techniques for synthetic polymers include solvent casting in conjunction with particulate leaching, phase separation, gas foaming, melt molding and fiber bonding [40, 43], all of which involve high temperatures, the use of chemicals or pH levels unsuitable for protein-based natural polymers. Consequently, the number of methods to generate pores in natural polymer is quite limited. Two of the most commonly used methods are freeze-drying [44] and critical point drying.

The use of 3D printing has gained considerable attention in recent years. This technique is especially fitting to generate OC scaffolds, since this tissue has a complex graded structure where biological, physiological and mechanical properties vary significantly over the full thickness of OC unit [45]. “Solid free form” technologies including 3D printing provide us with tools to closely control the design and shape (including the distinct curvatures of joints) in the final products; hence, producing tailorable scaffolds has become a reality. Different techniques of 3D printing are extensively discussed in [46, 47] and [40].These include direct 3D printing, indirect 3D printing [48] bioprinting (using a “bioink” or cell-laden gels)[49, 50], fused deposition modeling (FDM) [51], selective laser sintering (SLS) [52] and stereolithography (SLA).

### Recent advances in processing techniques for personalized osteochondral scaffolds

3D printing techniques are driving a shift toward personalization, as personal scans of joints can be converted into computer-aided design (CAD) files, which are then used to design anatomically accurate and patient-specific scaffolds and implants. Such personalized anatomical shape will secure a continuous transition between graft and host, contributing to an appropriate fit [53].

There are three broad approaches in 3D printing for TE applications: cell-free 3D-printed scaffolds, bioprinting of cell-laden scaffolds and bioprinting of scaffold-free constructs.

**Fig. 3:**
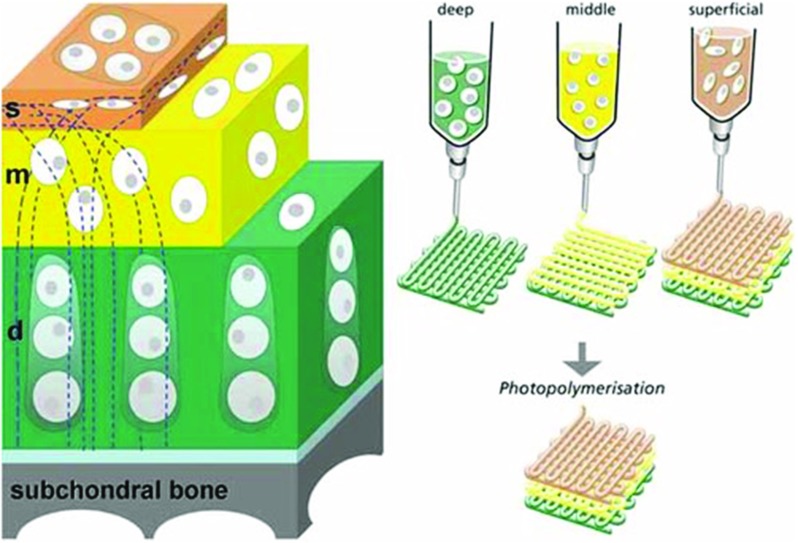
Concept for printing zonal osteochondral constructs where chondrocytes from the deep, middle and superficial zones are suspended in distinct hydrogel precursors and printed using bioprinting, from [95]. Reproduced with permission from JohnWiley & Son

Bioprinting, a process by which several types of living cells and biomaterials can be deposited simultaneously and precisely in a layer-by-layer manner [54], is especially promising to create OC scaffolds as it provides a way of recapitulating the heterogeneous and zonal architecture of the OC unit (Fig. 3).

Recently, a number of studies have explored whether bioprinting can be used to engineer cartilage tissues with regional distinctions in their composition [55], while a gradient in architecture (pore size) of printed polymer scaffolds was shown to alter cell distribution, although no influence on tissue composition was observed [56].

While bioinks and hydrogels used in bioprinting are favorable for mimicking the native ECM and providing an appropriate microenvironment for the cells, they are limited by a low compressive stiffness unsuitable for load-bearing applications. Incorporation of ultra-thin reinforcing fibers into hydrogels by melt electrospinning writing (MEW) may address this issue [53]. This technique allows precisely controlled deposition of these microfibers and has shown to generate structures with similar compressive behavior as native cartilage [57]. Incorporating cell-laden microcarriers has also shown to improve compressive modulus of bioprinted hydrogel construct. In one study, mesenchymal stem cell-laden PLA microcarriers were encapsulated in gelatin methacrylamide–gellan gum bioinks. This bioprinting approach allowed for the fabrication of constructs with high cell concentration and viability. Microcarrier encapsulation improved the compressive modulus of the hydrogel constructs, facilitated cell adhesion and supported osteogenic differentiation and bone matrix deposition by mesenchymal stem cells [58].

Bioprinting was used in a proof of concept study to regenerate the whole articular surface of rabbit synovial joint [59]. In this study, the surface morphology of a rabbit joint was captured with laser scanning and reconstructed by CAD. Based on that, anatomically correct bioscaffolds using a composite of poly-$\varepsilon$-caprolactone and hydroxyapatite, spatially infused with transforming growth factor $\upbeta$3 (TGF$\upbeta$3)-adsorbed or TGF$\upbeta$3-free collagen hydrogel, were fabricated and implanted in rabbit condyles. Four months after surgery, TGF$\upbeta$3-infused bioscaffolds were fully covered with hyaline cartilage in the articular surface [59].

Scaffold-free bioprinting is another promising approach to recapitulate tissue biology. In contrast to scaffold-based bioprinting, where tissue development depends on cell proliferation within the scaffold, scaffold-free bioprinting can offer relatively high cell density initially without the inclusion of biomaterials to facilitate the deposition of ECM in a defined manner [60]. However, bioprinting of scale-up tissues at clinically relevant dimensions is still a major challenge. To overcome this, a research group has fabricated scaffold-free scalable tissue strands as a novel bioink material. This microextrusion technique is capable of producing near 8-cm-long tissue strands (as opposed to 400 $\mu$m spheroid in traditional techniques).

### Clinical requirements of osteochondral Scaffold

**Fig. 4:**
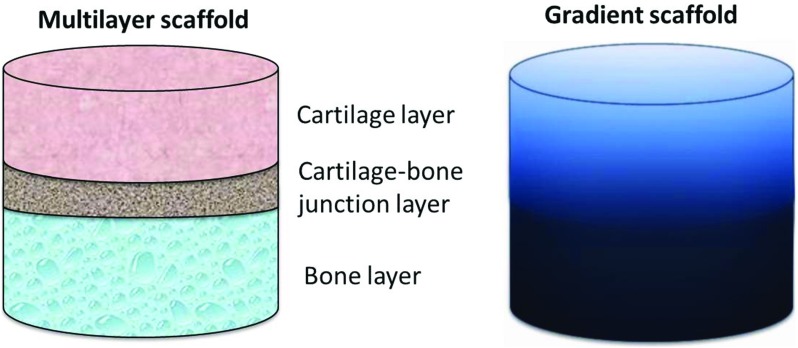
Schematic representations of multilayered and gradient OC scaffolds; in multilayered scaffold, each distinct layer corresponds to a layer of the native tissue, whereas in gradient scaffold the transition is gradual

Once implanted within the joint, the OC scaffold is exposed to a dynamic biomechanical host environment, along with changes in forces such as stresses, strains and fluid pressure. In order to achieve a heathy cartilage repair using multilayered scaffolds (Fig. 4), it is crucial for each layer to have mechanical properties that match the surrounding tissue and that the scaffold is mechanically stable to withstand the joint’s physiological loading without driving itself into fatigue or failure [61]. Hydrostatic pressure (HP) formed within the cartilage section has been perceived as one of the most important mechanical stimuli for chondrocytes and cartilage regeneration. As such, the OC scaffold should generate HP within the scaffold once implanted in to the osteochondral defects.

Degradation rate of OC scaffolds is also an important issue to ensure a balance between providing support for the cells and not restricting their growth and tissue formation. The scaffold should possess an appropriate biodegration rate that matches the new tissue formation, so that the scaffold materials could be gradually replaced by the newly formed tissues.

#### Mechanical properties

In healthy human articular cartilage, the tensile modulus measures at anywhere between 5 and 15 MPa, depending on whether the region of cartilage being measured is experiencing load of high weight or low weight [62]. Studies on the compressive behavior of articular cartilage reveal values of compressive modulus varying from 2 to 10 MPa shortly after application of load [62]. This, however, arises from interaction of cartilage aggregate with the fluid in the joint: The aggregate modulus (equilibrium compressive modulus) of native cartilage is 0.79 MPa and ultimate compressive strength is 7–23 MPa. In the joint, cartilage is typically exposed to stresses between 3 and 10 MPa, with stress as high as 18 MPa having been reported in the hip joint [63].

In terms of bone, mid-range values for the compressive modulus of cancellous bone are 90–400 MPa. However, it must be noted that the values of native bone vary considerably across different locations and patients. An example is the compression modulus of human cancellous bone obtained by Martens [64], where superior–anterior femoral head showed a modulus of 900 ± 714 MPa, while the anterior–posterior showed a modulus of only 12 ± 6 MPa and medial–lateral a modulus of 63 ± 7 MPa [64]. The appropriate target values therefore should be set based on the target location and should cover the stress range cartilage is exposed to.

The modulus for the calcified cartilage is more than an order of magnitude lower than the modulus of the underlying subchondral bone. This supports the idea that the zone of calcified cartilage forms a transitional zone of intermediate stiffness between the articular cartilage and the subchondral bone [65]. As such, the criterion for compressive modulus of this layer is set an order of magnitude lower than the bone section [66, 67].

#### Hydrostatic pressure for cartilage regeneration

Hydrostatic pressure (HP) is emerging as arguably one of the most important mechanical stimuli for cartilage and provides a robust method of chondrocyte stimulation [63]. In vivo, articular cartilage is exposed to a wide range of static and dynamic mechanical loads, ranging amplitudes of about 5–6 MPa for gait, and as high as 18 MPa for other movements such as running or jumping. In accordance with the biphasic model of cartilage, the solid components of the ECM support shear stress, whereas the incompressible interstitial water is responsible for withstanding compressive loading, by driving out of the tissue. In view of this, 95% of the overall applied joint load is supported by interstitial fluid pressurization, so HP is the prevailing mechanical signal governing normal articular cartilage homeostasis [68]. HP also appears to be useful in providing chondroprotective effects to chondrocytes subjected to an inflammatory stimulus. In addition to its wide use as an agent for mechanical stimulation in TE, there has been tremendous use of HP as a method of differentiating cells toward a chondrogenic phenotype [63]. However, raising pressures above these physiological levels has been shown to have limited or even detrimental effects

#### Biodegradation

TE scaffolds are inserted into the site of tissue damage, merely to provide support architecture for the development of new tissue, and so are required to degrade with time. This resorption is crucial once the scaffold has served its purpose, to avoid the risk of developing inflammation [69]. This degradation should occur naturally by the replacement of the three-dimensional structure, with the body’s own cells [27]. Facilitating regeneration of cartilage to begin with, requires that the implanted scaffold remains stable for at least two–three weeks. Stability of the scaffold in this period allows sufficient time for the composition of support structures for subsequent regeneration of tissues [70]. Scaffolds must possess flexibility in terms of their degradation following tissue regeneration. It is therefore crucial to select scaffold materials accordingly. For example, volumetric decreases in the PLGA scaffold were seen as quickly as 8 weeks following implantation, with majority of these structures losing their form following their absorption at 16 weeks [70], while a pure PLA scaffold resorbs after 1.5 years.

### Clinical osteochondral scaffolds: state of the art

A great number of scaffolds have been fabricated and explored for OC TE. Of those, only a small number has been advanced into clinical trials [71]. Examples are Biopoly, Chondromimetic, MaioRegen and Agili-C, which have been used for treatment of small cartilage and OC defects ($<1.5\,\hbox {cm}^{2})$ and are summarized in Table 1.

**Table 1:** Defect condition and patient characteristics of the current OC scaffolds in case of trauma and OA/OCD. Data from Clinicaltrials.gov

Scaffolds	Material	Defect size	Defect thickness	OA inclusions	Exclusions	Location	Age
Biopoly	Ti, hyaluronic acid and ultrahigh molecular weight polyethylen	$< 3.1\, \hbox {cm}^{2}$	$\,<$4 mm	ICRS grade 2, 3 or 4		Knee	Over 21
MaioRegen	Multilayered collagen–hydroxyapatite	2–9 $\hbox {cm}^{2}$		Chondral lesion of grade III/IV (outerbridge)	K-L$\,\ge \,3$	Knee	18–60
Agili-C	Hyaluronic acid+aragonite (cartilage)–aragonite (bone)	1–7 $\hbox {cm}^{2}$	$\,<$8 mm	ICRS IIIa–IVb	K-L = 4	Knee	Over 18
INSTRUCT					Severe OA		18–55
Chondromimetic	Collagen and glycosaminoglycans (GAG) and an osseus layer with collagen, GAG and calcium phosphate	$\le$ 12 mm	$\le$ 8 mm		Severe OA	Knee	18–65
BiCRI	Polylactic-co-glycolic acid (PLGA) and PLGA plus b-tricalcium phosphate	> 3 $\times$ 3 mm		ICRS grades 3–4 lesion, Outerbridge grade 4, or OCD grades 3–4		Knee (condyles/trochlea)	Up to 54

Current clinical scaffolds are mainly designed for the defects in knee joints and are largely aimed at adults with only one exception—BiCRI—which can be used in children. In addition to the age of the patient, the choice of a suitable scaffold also depends on the lesion size and condition of the disease. While Biopoly should not be used for lesion sizes exceeding $3.1\, \hbox {cm}^{2}$, MaioRegen can cover areas up to $9\, \hbox {cm}^{2}$. Severe OA in most cases is a contraindication for using these OC scaffolds.

The clinical performances of these scaffolds have been published in several studies [72–78] showing favorable results in terms of cartilage regeneration, pain reduction and regaining function. Most recently, however, a poor OC repair in 1 and 2.5 year postoperative assessments and incomplete regeneration of the subchondral bone was observed in MaioRegen [79]. The interface between the graft and the neighboring native bone as well as the boundary of the bony pit was still distinguishable after 12 months in BiPhasic scaffolds [72]. Chondromimetic combined with BMP-7 also led to the formation of subchondral bone cysts [80].

## Lessons from clinical osteochondral scaffolds and what we have learned from osteoarthritic joints?

The significance of subchondral bone integration in maintaining a healthy articular cartilage is well established [79, 81], especially from biomechanical and nutritive perspectives.

In general, during physiological loading, a range of mechanical forces is exerted on cartilage such as compressive and shear stress. These external stresses induce hydrostatic pressure in the cartilage and biofluid flow in and out of the cartilage. The function of subchondral bone is to support the overlying cartilage and protect the underlying cancellous bone from high stresses. Changes in the properties of the subchondral bone lead to increased strain generated in the cartilage layer, thereby initiating/maintaining matrix degradation, which can contribute to initiation/progression of OA [82, 83]. Delivery of oxygen and nutrition to different zones of articular cartilage takes place either through diffusion from synovial fluid or through diffusion from micro-blood vessels within subchondral bone depending on the zone of cartilage. Both diffusions are needed to maintain a healthy articular cartilage. Therefore, degeneration of cartilage in long run is expected if the support from subchondral bone is compromised, pointing to a possible reason for failure of healthy regeneration of cartilage as reported in the clinical studies.

**Fig. 5:**
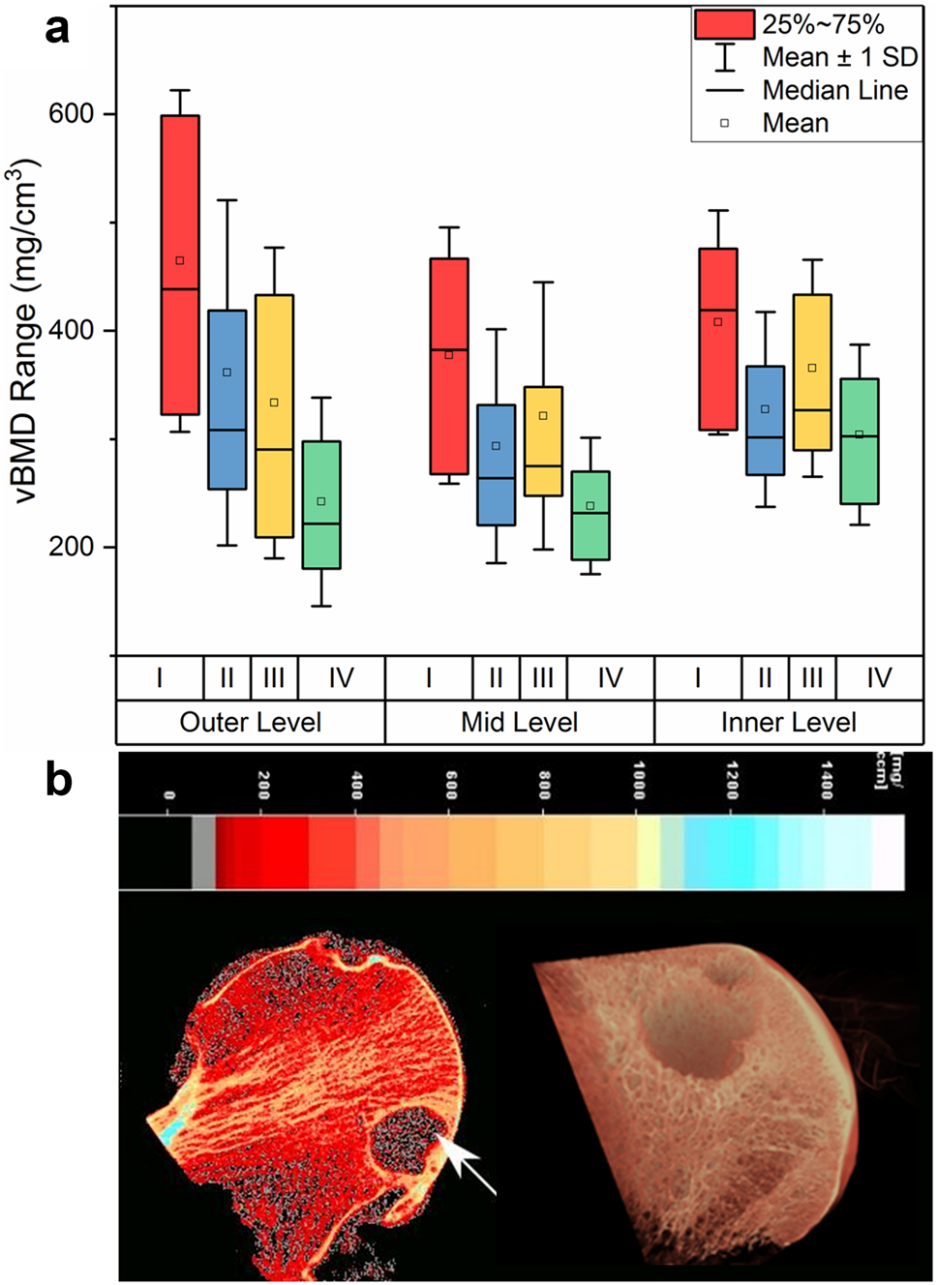
**a** Volumetric bone mineral density (vBMD) of OA samples according to both cartilage grading and depth; **b** presence of a large cyst (white arrows) in an osteoarthritis sample (pQCT image) [84] and microCT image

To develop an effective treatment for progression of OA, it is important to understand how the physical environment provided by the subchondral bone affects the overlying cartilage. To better understand the relationship between cartilage defect and subchondral bone changes in OA, we conducted a study on osteoarthritic femoral heads collected from total hip replacement operations and examined the volumetric bone mineral density (vBMD) distribution using peripheral quantitative CT (pQCT) [84]. We observed a significant decrease in vBMD, which co-localizes with the damage in the overlying cartilage. This was not limited to the subchondral bone immediately adjacent to the cartilage defect but continued in the layers below (see Fig. 5a). Another characteristic feature of the studies tissues was the presence of subchondral bone cysts with varied size in the subchondral bone, normally observed at regions of greatest cartilage loss (see Fig. 5b).

**Fig. 6:**
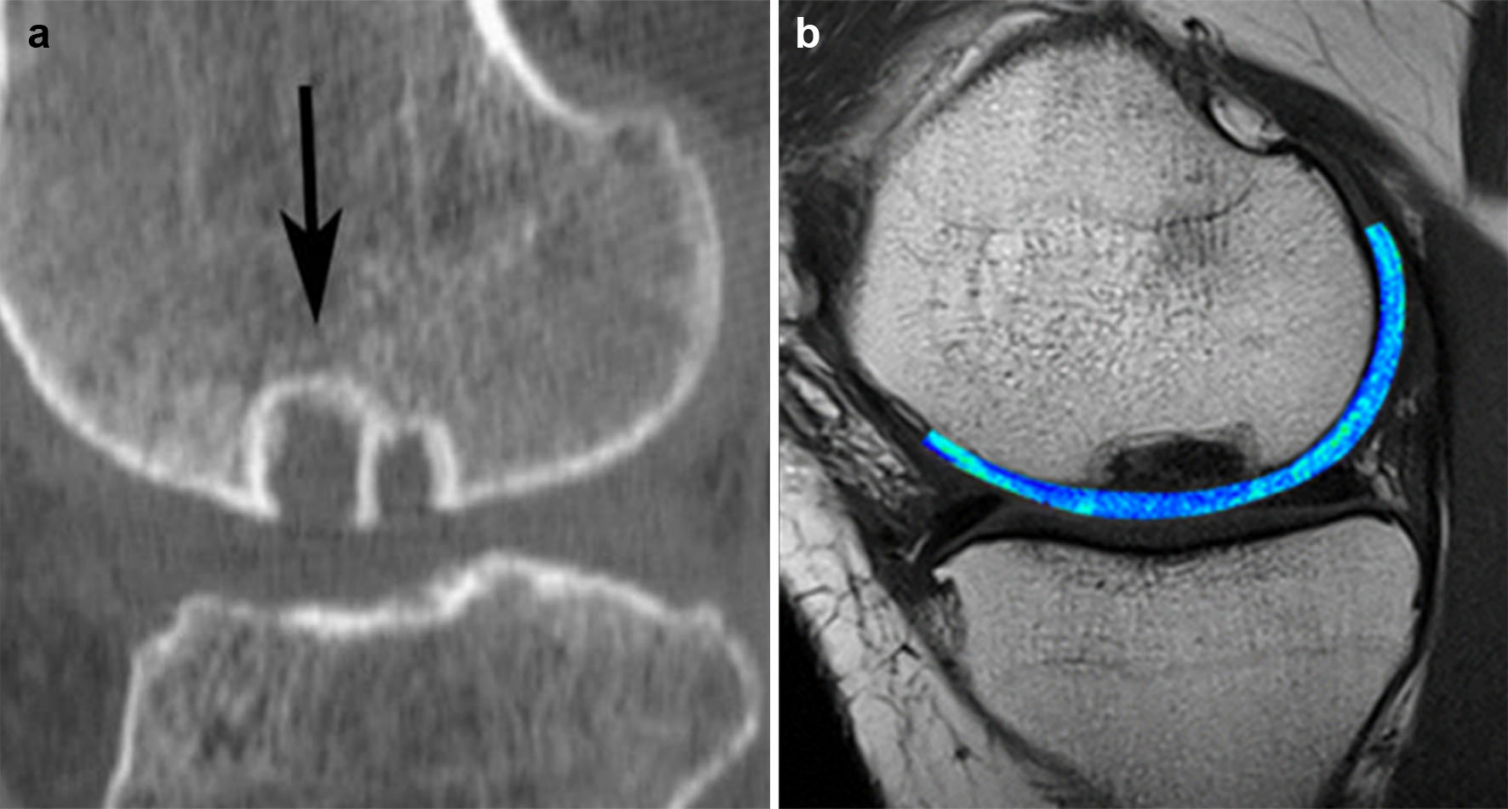
**a** CT scan failed to show bone ingrowth (*arrow*) into TruFit plug [90]; **b** T2 mapping MRI scan of OC lesion repair after 18 months by the use of the MaioRegen$\circledR$ scaffold, and bone cysts are observed [96]. With permissions from Elsevier and Springer

The cavities in subchondral bone, which are usually referred to as “subchondral bone cysts,” are normally reported in patients with OA. Usually, cysts observed in OA joints are in the range of 0.1–2.5 cm in diameter and appear in multiple. While smaller cysts are detected in the subchondral bone closer to the joint surface, larger cysts typically extend more deeply [85]. Subchondral bone cysts are recognizable in MRI images as areas of fluid signal and in radiographic images as lucent areas with sclerotic rims [86, 87]. The cysts observed in the terminal osteoarthritic cases in our study resembled those of “unfilled bone voids” observed in TruFit [88–90], MaioRegen and Chondromimetic (compare Figs. 5b and 6). It is possible that the existence of these cysts can lead to the changes in loading condition in the joint and affect the quality and durability of regenerated cartilage.

**Fig. 7:**
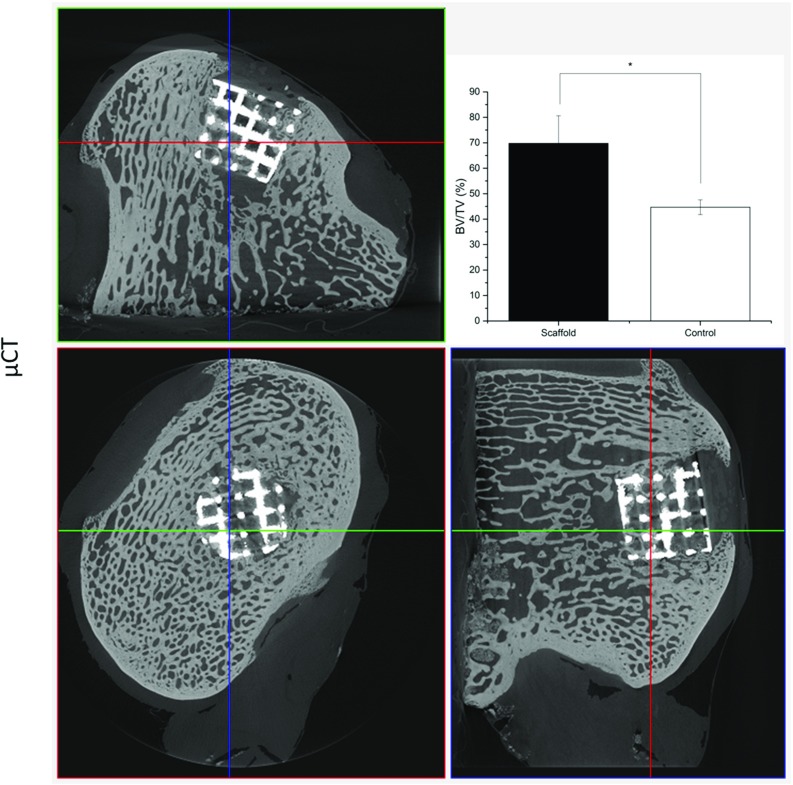
Bone ingrowth into Ti matrix. microCT images show bone formation within the Ti scaffold [94]

Although the primacy of the onset of articular cartilage degeneration and OA is still debatable [91], there is no doubt that the subchondral bone plays an important role in progression of the cartilage degeneration. In fact, there is evidence of communication, biomechanically and biochemically, between cartilage and subchondral bone. Where a healthy homeostatic cross talk leads to regulated bone remodeling and joint maintenance, a catabolic unhealthy cross talk leads to dysregulated bone remodeling and progressive damage [92].

As empirically observed in the commercial scaffolds, one of the dominant factors in suboptimum scaffold performance for cartilage regeneration seems to be the insufficient bone ingrowth and integration with the host tissues. Without a stable biomechanical support, the newly formed cartilage would “collapse.” The “collapsed” cartilage would not be subjected to mechanical stimulation [93], which is a critical factor for hyaline cartilage formation. As a result, poor cartilage fill and associated fibrocartilaginous repair rather than the hyaline cartilage, as well as poor OC repair, are often observed.

Considering the importance of a mechanically stable support for cartilage regeneration, we have recently developed an OC scaffold based on a multilayered composite system comprising a highly porous titanium base to encourage bone formation and provide support for the overlying cartilage. The porous titanium layer was produced by selective laser sintering from commercially pure titanium powder (cp-Ti) using a direct metal laser sintering system which resulted in a cylindrical scaffold with strut thickness of 0.5 mm, pitch size of 0.75 mm, porosity of 72% and mechanical properties in range of trabecular bone (compressive strength 35 MPa and modulus 73 MPa). Bone formation and ingrowth into the titanium scaffolds were evaluated in sheep stifle joints. The examinations after 3 months revealed 70% bone ingrowth into the scaffold as shown in Fig. 7, confirming its suitability to be used as a stable support for cartilage regeneration [94].

The scaffold has also been tested in a clinical dog shoulder model where an OCD had occurred due to natural development of OA in the dog. The 3-month follow-up arthroscopic examination revealed the cartilage had regenerated well, matching the curvature of the joint perfectly. Recent reports from the dog owner suggested the dog shoulder function was recovered completely. A glimpse of how this scaffold will perform has been given, with promising results, by Professor Noel Fitzpatrick of the Channel 4 TV series Supervet.

This biomimetic OC scaffold has the strength needed to bear the physical load of the joints and its biomechanical structure encourages consistent cartilage fill and a smooth articular surface. It has the potential to address the unmet clinical need for repair of large OCDs. This functional biomimetic OC scaffold bridges the gap between small OCD treatment and joint replacement. It is hoped that it will provide clinicians with a viable treatment option in situations where the disease has progressed beyond a small defect, but where a full joint replacement could still be avoided.

## Perspective summary

OCDs typically derived by traumatic injuries or OA involve articular cartilage and associated subchondral bone. These defects are characterized by unbalanced degeneration and regeneration of articular cartilage and bone where the intrinsic repair mechanisms are insufficient. Stopping or delaying progression of OCDs would have significant impact on health care.

The treatment of cartilage and OC defects remains a challenge because treatments to date have failed to achieve a complete restoration of the joint cartilage surface and its properties. Many new technologies, such as OC TE and stem cell therapies, have been studied and applied to the repair of OC defects. The goal of a TE approach is to repair the defect in the joint and restore its function in order to delay or remove the need for a joint replacement.

Numerous OC scaffolds have been developed by different research groups around the world, and there are many commercially available products. However, few of these products promote satisfactory durable regeneration of large OC defects. The authors believe that the subchondral bone and adjacent cartilage form a functional unit where the subchondral bone is critical for the successful repair of cartilage and OC defects. Lessons learned from the clinical trials suggest that an improved biomechanical fixation of the OC scaffold would provide an appropriate physical environment for healthy growth of the overlying cartilage.

Development of a functionally biomimetic OC scaffold which will bridge the gap between small OC defect treatment and joint replacement is still a grand challenge. However, with the advancing of OC scaffold biotechnology, it is hoped that, in the near future, a novel OC scaffold with improved capability for biomechanical and biological fixation would lead to tangible and clinically relevant results in a one-step surgical procedure for the treatment of large OCDs, relieving pain and improving quality of life by keeping people active.
